# Hospital and Institutional News

**Published:** 1918-04-06

**Authors:** 


### HOSPITAL AND INSTITUTIONAL NEWS.

### CURATIVE WORK IN THE EASTERN COMMAND.

THE King paid a visit last week to the Command Depôts of the Eastern Command, where many thousands of officers and men are undergoing special treatment, mainly in the form of massage and curative exercises. Much interest was displayed by His Majesty in the round games—handball and the like—organised to bring atrophied limbs again into use. Some of the more advanced cases are able to undertake exercises of quite a strenuous character, and the King witnessed some very clever and amusing boxing bouts. Allotment gardens are a feature of the scheme, this form of exercise being especially valuable in shell-shock and neurasthenia. Visits by relatives are encouraged within the limits of the practicable, for which purpose "Welcome Huts" are at the disposal of officers and men in which to receive their friends.

### THE MILLION HALF-CROWNS.

HIS MAJESTY THE KING has given great impetus to the London Hospital Half-crown Appeal by a donation of a sum representing 4,000 half-crowns, and the Lord Mayor has interested a small but select party of twenty donors, who have each contributed £1,000. If the half-way house of the million has not yet been reached it cannot be far away, though it is the second moiety of a large sum in such an appeal as this that takes the getting. But Lord Knutsford does not know the word "failure," and if he has collected the first half of his requirements in the first quarter of the year, the success of his great offensive before December 31 seems to be assured. Was it not the trustees of Sir William Dunn's estate who brought the last quinquennial appeal to a triumphant conclusion? Perhaps history will repeat itself, and some other large donor will lay the coping-stone. The legacy does not, of course, affect the Half-crown Appeal, but we see that Mr. James Hora has bequeathed the residue of his estate, which will exceed £100,000, to the Samaritan Fund of the London Hospital. Mr. Hora, who was a director of several Colonial and other companies, named Viscount Knutsford, chairman of the London Hospital, as one of his executors.

### A GROUP OF NERVE HOSPITALS FOR PENSIONERS.

AT the request of the Ministry of Pensions, the National Hospital for the Paralysed and Epileptic, Queen Square, has undertaken extensive provision for discharged disabled sailors and soldiers suffering from the special class of diseases treated by the hospital. Two large houses in Queen Square are being converted into an annexe of the hospital containing thirty beds for discharged men. The cases will be primarily chosen from the few patients who suffer from both epilepsy and paralysis, and the remainder of the beds will be devoted to various forms of paralysis. The Board has also assumed the management of Lonsdale House, Clapham Park, a hospital of twenty-five beds for discharged paralysed sailors and soldiers. This institution, completely equipped, was handed over to the National Hospital on February 1. Lonsdale House is adapted to its present purposes and stands in extensive grounds. Later the hospital will also take under its management a Home of Recovery for neurasthenic discharged men, which will probably be organised similarly to the home at Golders Green, managed for the Ministry of Pensions by the Hospital for Epilepsy and Paralysis, Maida Vale. Negotiations are in progress to acquire for the purpose a large house and estate about twenty-five miles from London, suitable for the treatment and training of 100 patients. Thus, on behalf of the Ministry of Pensions, the hospital will administer three separate hospitals, containing in all about 160 beds.

### THE PROMOTION OF MAJOR RHODES.

THE North Staffordshire Infirmary sustains a serious loss in the promotion of its able and popular secretary-superintendent, Mr. T. Basil Rhodes, to a post in the Treatment Branch of the Ministry of Pensions. Major Rhodes (as he has become since the infirmary added on a war hospital) was the first medical man to perform the dual functions of secretary and house governor at Stoke, and he has succeeded to admiration in uniting the medical and the business control of the institution. He has done good work also in the new military branch of the institution, and is one of those men marked out for promotion by reason of high administrative gifts. The infirmary is to be congratulated on having held him for eight years, and his work there will endure, for it is of the kind which has its fruition in the future, though the immediate reward of public appreciation and the regard of colleagues is not wanting. Major Rhodes has been selected to work under Lieutenant-Colonel Sir John Collie, M.D., C.M., R.A.M.C., at the problems relating to the accommodation in hospitals of discharged sailors and soldiers, the general facilities available for their hospital treatment, and the wider questions relating to hospital provision in regard to the needs of the civil population. The country needs for such offices men of sound constructive ability, who, while concentrating on the paramount necessities of the sister Services at the moment, have yet an eye to national conditions when war is over. In choosing Major Rhodes the authorities seem to have got hold of the right man. His successor at the infirmary will find his path considerably smoothed by the good traditions established in the secretariat.

### CIVILIAN BEDS AND THE GREAT OFFENSIVE.

THE recent occurrence of two cases of measles in the Gulson Ward of Coventry and Warwickshire Hospital, which has led to its temporary closing, has helped to swell a long waiting-list. In fact, the demand of civilians upon the hospital's beds grew so acute that it resulted in the release by the War Office, at the request of the Ministry of

Pensions, of certain beds ordinarily reserved for wounded soldiers. This arrangement, which more or less coincided with the German offensive in France, leads one to wonder what the situation might have been, not only at this hospital, had not the British withdrawal doubtless necessitated the leaving of many of its wounded in German hands. While probably this fact accounts for the numbers of prisoners which they claim (a number which so regarded must be considered relatively small), it does remind us that an emergency may very quickly fill the reserve beds which are often temptingly empty. In the case of a British offensive, when few or none of our own wounded are taken prisoner, a general hint some weeks in advance may suffice. But when by an accident like that at Coventry one or more of the civilian wards are suddenly rendered useless, the waiting-list may become more than a problem. It may develop into crisis. This being so, it seems to us that voluntary hospitals should fix with care the limit beyond which they cannot go in the admission of military patients; and that the War Office, so far as possible, should prefer additional institutions, or specially added wards in voluntary hospitals, to the extensive adoption of reserving beds in the civilian wards of the latter.

### A NEW DEPARTURE AT THE CANCER HOSPITAL.

The Earl of Northbrook, who presided at the recent annual meeting of the Cancer Hospital, in noting the decrease in patients, remarked that it had been the experience of all hospitals in regard to civilian patients since the outbreak of the war. But he was a little surprised that the number of out-patients showed a decrease, because they had had a number of military patients for nervous disorders, or for the effects of bullet wounds. He had expected that those military cases would have made up for the falling off in the number of their ordinary patients. The committee was much gratified at the conferring upon Dr. T. J. Harder of a knighthood in recognition of his research in bacteriology. It is satisfactory to learn that since the rationing orders the difficulties of obtaining supplies for the hospital had very considerably diminished, and they were now able to obtain the supply of food to which they were entitled. A new departure in radiation work had been inaugurated by Dr. Knox and Mr. Percival Cole, who collaborated in treating soldiers suffering from deformities of the face, the result of gunshot wounds, which in turn led to the formation of keloid contractions. A way of removing these curious scars has long been sought without success, and the results of the above plan are worthy of record.

### THE ROBERT BURNS COTS: A SUGGESTION.

The Salford Royal Hospital has received the presentation of a cot endowed in memory of Robert Burns, from the Burns-Scott Memorial Fund, a gift which has been gratefully acknowledged by Dr. Hopkinson, the hospital's treasurer. This particular endowment is the fruit of a legacy from the late Mr. J. T. Blair, but similar endowments have been given to St. Mary's Hospital and Ancoats Hospital, Manchester. In commending the generosity of the fund, we should be glad to know how hospitals may qualify to receive these endowments, and whether the Burns-Scott Memorial Fund was founded to perpetuate the memory of these men of letters mainly by endowing beds in remembrance of them. If Mr. William Thompson, who made the presentation to the Salford Royal Hospital, will explain these points, not only would other hospitals be interested, but the success of his fund might lead to the creation of others to perpetuate, by the endowment of hospital beds, the memory of other poets and writers. The matter is interesting, since admirers of men of letters do not generally choose philanthropy or charity as the expression of their admiration. At least, so far as we are aware, the Browning Society does not endow hospital beds, nor do the Wordsworth or Shelley Societies subscribe to charity. Though these do not describe themselves as memorial societies, their activities have the effect of a memorial. Does the Burns-Scott Memorial Fund perpetuate the memory of these by endowments only?

### A HOSPITAL SECRETARY'S LEGACY.

It is pleasant to record the legacy of £20 which has been received by the Colwyn Bay and District Hospital from the executors of the late Mr. James Wood. Not only was Mr. Wood one of the founders of the institution, but he acted for many years as its honorary secretary. Legacies to institutions by their administrative officers can never be very numerous, and they are always, therefore, peculiarly touching.

### A WAR MEMORIAL AT TUNBRIDGE WELLS

Sir David Salomons, who provided the General Hospital, Tunbridge Wells, with a new electrical department in memory of his only son, Captain Salomons, who was drowned off the Gallipoli Peninsula when H.M.S. *Hythe* was sunk after a collision, has now formally opened the department, which is nearly complete. The original installation was Sir David's gift, and has been regularly in use for some time.

### A BATTALION'S REMARKABLE GIFT.

The Institutes Committee of the 21st Battalion King's Royal Rifle Corps (Yeoman Rifles) has given £50 to the York County Hospital on condition that a bed should be named to perpetuate the memory of the battalion. The original members of the battalion, which was raised in September 1915, were all Yorkshiremen, and the present officers and men wish that "the existence of a battalion containing such a preponderance of local residents should be kept alive in the heart of the city." In enclosing the cheque and making the request, Lieutenant-Colonel G. L. Brown remarked "the amount is not large, but I feel that you will realise that the spirit which has prompted the gift is one which cannot be measured in money." The secretary, Mr. F. Neden, in acknowledging the gift, drew attention to the rule that a sum of £1,000

was required to endow a bed, but added that he was sure his committee would be happy to place a memorial in the hospital to the battalion, and asked if an inscription over one of the beds in the men's surgical ward would be acceptable to the battalion. We applaud the spirit which prompted the gift and its acceptance.

### THE FRUITS OF GOOD WORK AT CHESTERFIELD.

For the first time for twenty years the Chesterfield Royal Hospital is out of debt, and this satisfactory achievement has no doubt influenced the authorities in granting permission for the use of the above addition to its title. The removal of the debt has been secured by the efforts of the local traders, who raised £8,300. Half of this paid off the debt, and the remainder is being put aside for expenditure on improvements and extensions after the war. It is noteworthy that the total receipts for the first time have reached over £20,000, and that rather more than half of this sum is represented by workmen's contributions. Mr. E. C. Barnes, who has been the hospital's chairman for eighteen years, lately spoke of the assistance which the workmen's representatives had given to the committee as much in administration as in financial help. We hope that the recognition which the hospital's work has won will start it upon a new extension of usefulness.

### THE PENALTY OF INEXPERIENCE.

At a meeting of local subscribers to the Priestley Green Auxiliary Hospital, Lightcliffe, the dispute between themselves and the commandant, Lady Firth, which has led to the temporary closing of the hospital, was discussed in public. The chairman of the committee, Mr. J. G. Mallinson, presided, and Sir Algernon Firth, who was represented by his legal adviser, Mr. J. F. Hirst, was present. Mr. Mallinson explained that £1,200 had been subscribed for the equipment and maintenance of the hospital on the understanding that a committee elected by the subscribers should undertake the whole responsibility of management and finance. But since Lady Firth was appointed commandant she had entirely controlled matters, to the exclusion of the committee. Mr. Hirst, in reply, said that the trouble arose because the first public meeting included no one who knew anything about the management of auxiliary military hospitals. The hospital belonged to the St. John Ambulance Association, and Lady Firth, as commandant, must have done what she did. The committee's only function was to administer the funds which its members raised, which were a fraction of the sum received from the Government. In the end a vote of confidence in the committee was passed by a large majority. But Sir A. Firth said the hospital would be reopened, and subscribers would know the character of the management under the rules of the St. John Ambulance Association.

### THE PATIENTS OF ST. DUNSTAN'S.

The third anniversary of the opening of St. Dunstan's Hostel was celebrated by a presentation to Sir Arthur Pearson of a silver casket, which was handed to him by Mr. Ernest Kessell on behalf of past and present patients. In acknowledging the gift, Sir Arthur attributed the success of the institution to the absence of red-tape and official interference, in spite of the splendid co-operation which had been given by the War Office, the Army Council, the R.A.M.C., and the Red Cross. The public had been most generous, and he mentioned that for the Children's Fund of the Hostel £125,000 had been subscribed in the past six months, which led him to believe that the total sum required—namely, a quarter of a million—would eventually be forthcoming. It is interesting to learn that, of the 1,100 men who have passed through St. Dunstan's, and there are 569 there at present, only one has refused to take advantage of the training. In being able to state that most of the men who had left were earning, apart from their pensions, more than when they could see, we hope that Sir Arthur is assured that these profitable occupations are likely to survive the troubles of the labour market after the war. This point is so important that his assurance and the evidence on which it is based would be very welcome.

### THE DANGER OF THE UNVACCINATED.

The outbreak of small-pox, fortunately as yet numerically negligible, has called public attention once more to the danger arising from the "conscience" clause of the Vaccination Act. The number of exemptions so granted has, as the Medical Officer of Health for Surbiton pointed out in the *Times* the other day, steadily increased, till in 1913 (the latest date at which exact figures are available) exemptions were being granted at the rate of 296,000 per annum, representing a proportion of some third of the total births. Moreover, there is every reason to believe that the numbers have been on the up grade since that year. Many of the exempted children are now at school, and the appalling results that would follow an outbreak can be better imagined than described. The Local Government Board have, we understand, issued a circular letter to Boards of Guardians emphasising the necessity for prompt measures to deal with the vaccination and re-vaccination of persons who have been in contact with infection, and are willing to sanction the payment of fees for them, even if they are not actually resident in the district where the vaccination has been performed.

### NEW SCHEME FOR SOCIAL WORKERS.

On November 9, 1917, a conference was held at the London School of Economics to consider the need for the organisation of social workers. This conference was the result of some articles that had previously appeared in the *Times* dealing with the question. At the conclusion of the conference it was agreed to appoint a provisional committee representative of both voluntary and professional social workers, which committee was to consider what steps might be taken to promote such organisation. This committee has since met frequently and has attempted to draw up a suitable scheme. It is thought that social workers should form themselves into groups according to the work in which they are engaged; that these groups should discuss and decide their own particular problems, and make what regulations, demands, and suggestions in regard to training, salaries, and general conditions of employment they think fit. Further, that in those fields of social work in which many volunteers are employed, these voluntary workers should form similar associations. Once such associations are formed each should be represented by elected delegates on a Central Council, which Council would deal with such questions as pertain to social work in general. Broadly speaking, this is the very pliable structure which the committee proposes as the basis of organisation. Before venturing further it is necessary to know whether social workers as a whole feel the need for organisation. If the work of the committee is to continue, funds will be necessary in order to cover the initial expenses that such a movement must necessarily entail. It is therefore hoped that those who are interested will show such interest by sending small donations of 1s. or more to Miss Nora Milnes, the hon. secretary to the provisional committee, at the London School of Economics, Clare Market, Kingsway, W.C. 2. The honorary secretary will be glad to furnish any further particulars that those who feel such interest may desire.

### NURSES AND THE MEAT RATION.

In spite of the ordinary assistance given by local Food Committees, some hospitals have feared or found that the meat allowance was insufficient for their needs. At a meeting of the Queen's Hospital, Birmingham, Mr. Charles Hyde lately declared that, in view of their long hours of work, it was essential for nurses to have more meat than was supplied to ordinary sedentary workers. He also claimed similar relief for patients who were recovering from long and serious illness. In respect of the latter, Mr. Hyde has gained the support of the Lord Mayor of the city, who has promised to approach the local Food Committee. Now that the rationing scheme has been applied, and the distribution of meat has been evenly, if modestly, carried out, there should be an opportunity to place the needs of hospitals and nurses before the Food Committees with some chance of a reasonable hearing. In some areas, at all events, there seems more than enough to supply the rations, and, after proper regard for the future, there should be a sufficient margin to allow the needs of special workers to be met.

### CO-ORDINATION IN TREATMENT OF LIMBLESS CASES.

The scheme for the treatment and training of limbless sailors and soldiers now in operation in connection with the Welsh Metropolitan War Hospital, Whitchurch, Cardiff, is an admirable example of co-ordination of effort between a central hospital, an auxiliary Red Cross hospital, and an affiliated special hospital. In the first instance, all orthopædic cases go to the Welsh Metropolitan War Hospital, Whitchurch, for treatment or operation. In the case of limbless men, as soon as the stumps are fit, temporary artificial limbs (Belgian pylons) are made, and the patients are transferred to the Red Cross Hospital, Penarth, a seaside resort about six miles distant. Here, owing to the hilly character of the roads, legless men have ample opportunity for testing the temporary limbs and in this way assist the stumps to become set and firm. The sea baths at Penarth are also found of great benefit in the strengthening and hardening of the stumps. When a man is ready to be fitted with his permanent limb he is sent to the Prince of Wales' Hospital, Cardiff, where the artificial legs and arms are made and where the patient is properly trained in their use. On being passed out from the latter hospital, the men are re-transferred to Penarth or sent to one of the other auxiliary hospitals, awaiting discharge from the Navy, Army, or Air Service, as the case may be.

### A NEW "GAZETTE."

Netley Red Cross Hospital has joined the ranks of those which possess gazettes, and its first number has triumphed over all difficulties of paper shortage. Inspired by the suggestion of Sister Harvey, and edited by the Rev. Cæsar Caine, C.F., the first number runs to twenty-four pages. The elaborate Christmas entertainments live on in the illustrations, and suggest such an amount of talent and enterprise that the new magazine should soon collect a brilliant staff of writers, draughtsmen, and photographers.

### THIS WEEK'S DRUG MARKET.

Business has naturally been quiet during the past week owing to the Easter holidays. Activity, however, will be resumed almost immediately, as, in view of the extraordinary conditions prevailing in the market, it is necessary for buyers to watch the movements carefully from day to day. It now seems almost certain that a maximum price for saccharin will be fixed by the Food Controller, and this price will probably be less than half the price now ruling. The price of cocoa-butter has been fixed at a figure considerably below that at which business was being done prior to the intervention of the authorities. Senega-root is practically unobtainable on the open market; cascara sagrada is scarcer and dearer—in fact, all American drugs are likely to continue to advance in price in consequence of the new restrictions on imports. The demand for bromides is somewhat quieter, but prices are very firmly maintained, with a strong probability of a further advance. The scarcity of camphor has become more acute, and there is little doubt that the rise in prices will continue. Hexamine, acetanilide, aloin, trional, methyl salicylate, santonin, sulphonal, sodium benzoate, and clove oil are all dearer. On the other hand, caffeine, phenacetin, phenazone, and paraldehyde have an easier price-tendency at the moment. Reports from the Norwegian cod-fishing districts are to the effect that, up to the present, this season the catch has been abnormally poor, but as very little of the cod-liver oil is likely to reach this market interest in the fishing is lacking.

